# The Principles and Practice of Bandaging

**Published:** 1893-07

**Authors:** 


THE PRINCIPLES AND PRACTICES OF BANDAGING, by G. G. Davis, M. D., Detroit, Mich. Geo. S. Davis. Price, $3.00.

An excellent little book, published in good style and profusely illustrated, showing method of applying every style of bandage.

